# Head Gesture Recognition Combining Activity Detection and Dynamic Time Warping

**DOI:** 10.3390/jimaging10050123

**Published:** 2024-05-19

**Authors:** Huaizhou Li, Haiyan Hu

**Affiliations:** 1 College of Building Environmental Engineering, Zhengzhou University of Light Industry, Zhengzhou 450001, China; 2 Engineering Training Center, Zhengzhou University of Light Industry, Zhengzhou 450001, China; 2018850@zzuli.edu.cn

**Keywords:** head gesture, activity detection, dynamic time warping, inertial measurement unit, human–computer interface

## Abstract

The recognition of head movements plays an important role in human–computer interface domains. The data collected with image sensors or inertial measurement unit (IMU) sensors are often used for identifying these types of actions. Compared with image processing methods, a recognition system using an IMU sensor has obvious advantages in terms of complexity, processing speed, and cost. In this paper, an IMU sensor is used to collect head movement data on the legs of glasses, and a new approach for recognizing head movements is proposed by combining activity detection and dynamic time warping (DTW). The activity detection of the time series of head movements is essentially based on the different characteristics exhibited by actions and noises. The DTW method estimates the warp path distances between the time series of the actions and the templates by warping under the time axis. Then, the types of head movements are determined by the minimum of these distances. The results show that a 100% accuracy was achieved in the task of classifying six types of head movements. This method provides a new option for head gesture recognition in current human–computer interfaces.

## 1. Introduction

Enabling machines to understand the meaning behind human behavior has become one of the research topics that has aroused great interest among researchers. In particular, head gesture recognition plays an important role in human–computer interfaces. Currently, the main methods for detecting head movements include image processing, depth sensing, radar technology, and inertial sensors [1]. Head movement detection has a wide range of applications in various fields, such as human–computer interactions [2,3,4], assisted driving [5,6,7], virtual reality [8,9,10], augmented reality [11,12], and more.

Compared to image processing methods based on video and depth maps, an inertial measurement unit (IMU) sensor-based detection system has lower complexity, a faster processing speed, and a lower cost. Many researchers have studied algorithms for identifying head movement types using IMU sensors. To address the human–computer interaction problems of patients with quadriplegia, Rudigkeit et al. [4] achieved a maximum action recognition rate of 85.8% with an IMU sensor-based behavior-assisted robot controlled by head movements. Wang et al. [3] proposed using a simple nod of the head as a method for unlocking, in order to address the inconvenience caused by the reliance on external input devices for password authentication during the use of AR/VR devices. They selected initial-end postures as well as gesture dynamics, including the angular velocity magnitude, trajectory properties, and the power spectral density of the linear acceleration magnitude, as features to train a random forest classifier, achieving an accuracy rate of 97.1%. Severin et al. [13] conducted a series of studies on head movement classification using a 6DOF inertial sensor consisting of three-directional accelerations and angular velocities. Firstly, they compared various methods, such as random the forest classifier, the k-Neighbors Classifier (kNN), the Decision Tree Classifier, Gaussian Naive Bayes (NB), Logistic Regression, the Extra Trees Classifier, a Quadratic Discrimination Analysis, the Adaptive Boosting Classifier, and a Support Vector Machine (SVM), and achieved the best classification accuracy of 99.48%. Analyzing the impact of features on classification results, it was found that using 16 features represented by the dominant frequency, the max value of the time series, the min value of the time series, the time series length, the interquartile range, the median value, the mean value, autocorrelation, standard deviations, Kurtosis, autoregressive coefficients, energy, the Skew value, variance, Spectral Entropy, and Sample Entropy had better classification results than using six features. The former achieved a complete and correct classification except for the kNN algorithm [14]. When analyzing the impact of the number of sensors on classification results, it was found that a system with three sensors can achieve better classification results [15]. However, in an experiment, the three sensors were installed in completely different locations compared to the single sensor, which makes it difficult to draw a significant comparison between the two. Wong et al. [16] developed a smart helmet for motorcycle riders using an IMU sensor. They used a linear discriminant analysis and a neural network to recognize head movements with an accuracy of 99.1% and found that gyroscope sensor data are more suitable for head movement recognition than accelerometer data. It can be seen that significant progress has been made in the accuracy of head movement recognition, but high accuracy requires relatively more head movement time series features. The goal of this paper is to find a method that can still achieve completely correct classification results with fewer features.

Dynamic Time Warping (DTW) measures the similarity between two time sequences, which might be obtained by sampling a source with varying sampling rates or by recording the same phenomenon occurring with varying speeds. It has a wide range of applications in speech recognition and action recognition [17,18,19,20,21]. However, there are few studies on the application of DTW in head gesture recognition. Mavus et al. [22] were the first to study this and achieved an accuracy of 85.68% with only four types of head movement classifications, possibly due to the use of fewer features. Then, Hachaj et al. [23,24] trained a DTW barycenter averaging algorithm using the quaternion averaging obtained from IMU sensors and a bagged variation of the previous method that utilizes many DTW classifiers that perform voting. The classification results achieved an accuracy of 97.5%. This algorithm requires the average of a quaternion-based signal and the estimation of head movement trajectories for recognition, and it does not take into account the issue of endpoint detection for head movements.

Compared with previous studies, this paper achieves better classification performance by minimizing the use of feature numbers and reducing data processing. Furthermore, an activity detection algorithm for head movements is proposed to automatically extract time sequences of head motions instead of manual segmentations, which enables the adoption of this algorithm in devices such as VR/AR, wheelchairs, and assistive robots to help people improve their gaming experience or quality of life.

## 2. Related Work

### 2.1. Applications of Head Motion Recognition

Head motion recognition has been studied extensively over the years, and research results have been successfully applied in many domains. Rudigkeit et al. [4,25] developed a behavior-assistive robot that uses head gestures as a control interface. They found that the mapping of head motions onto robot motions is intuitive and that the given feedback is useful, enabling smooth, precise, and efficient robot control. Furthermore, Dey et al. [26] developed a smart wheelchair using IMU-based head motion recognition, enabling quadriplegic patients to easily navigate a wheelchair using only their head. Dobrea et al. [27] also successfully used a four-capacitive-sensor system to recognize head motions for wheelchair control. As AR/VR devices become increasingly prevalent in our lives, Wang et al. [3] developed a seamless user-authentication technique for AR/VR users based on biometric features extracted from IMU-sensor readings in the devices, using simple head motions. To identify the head movements of motorcycle riders, Wong et al. [16] also used IMU sensors and a neural network to recognize four types of actions (Looking Up, Looking Down, Turning Left, and Turning Right). They found that the head movements were concentrated on rotations, and the gyroscope data had greater importance than the accelerometer data. To facilitate smart watch users to accept or reject operations, Yang et al. [28] developed a head shaking and nodding recognition system based on millimeter-wave sensors. These research findings have provided possibilities to facilitate people’s work and life, especially for those with disabilities who cannot communicate through their limbs. This further demonstrates the important significance of in-depth research on head movement recognition.

### 2.2. Methods for Head Motion Recognition

Head motions carry a wealth of information that can effectively support and assist human communication. Ashish et al. [29] used an infrared-sensitive camera equipped with infrared LEDs to track aa pupil and used the pupil movements as observations in a discrete Hidden Markov Model (HMM)-based pattern analyzer to detect head nods and shakes. Their system achieved a real-time recognition accuracy of 78.46% on the test dataset. Fujie et al. [30] used the optical flow over the head region as a feature and modeled them using HMM for recognition. They used the optical flow of the body area to compensate for swayed images, as it was challenging to collect sufficient data to train the probabilistic models. They introduced Maximum Likelihood Linear Regression (MLLR) to adapt the original model to camera movements. Wei et al. [31] employed Microsoft Kinect and utilized discrete HMMs as the backbone for a machine learning-based classifier. They trained three HMMs (nodHMM, shakeHMM, and otherHMM) based on five head movement states (Up, Down, Left, Right, and Still), achieving an 86% accuracy on test datasets. Wang et al. [3] reconstructed head motion trajectories using IMU data from AR/VR devices and extracted features such as initial-end postures, skeletal proportions, and gesture dynamics for a random forest classifier in N-label classification tasks, achieving an average accuracy of 97.1%. Mavus et al. [22] proposed a genetic algorithm-based cost function minimization, but did not specify the exact IMU data used, achieving an accuracy of 85.68%. Hachaj et al. [24] trained a DTW barycenter averaging algorithm using the quaternion averaging obtained from IMU sensors, as well as a bagged variation of the previous method that utilizes multiple DTW classifiers with voting. Their classification results achieved an accuracy of 97.5%. Severin et al. [13,14,15] tested some classification methods based on IMU data and found that when using 16 features from the head motion time series, most classifiers can achieve a completely accurate classification. However, these studies did not cover the endpoint detection methods for head motion time series data, and endpoint detection is a necessary step for the real-time segmentation of head motion time series data.

This paper proposes a method for the automatic detection of head motion endpoints to extract time series data during head movements. Subsequently, using a small number of features, only involving the acceleration and angular velocity from an IMU, it analyzes the time series features of six common head motion types and creates templates for their motion time series. Then, based on the templates and the extracted head motion time series, the type of head motion is determined by calculating the shortest path using the DTW method, which has significant advantages in assessing the similarity of time series.

## 3. Material and Methods 

### 3.1. Data Acquisition

**Figure 1:**
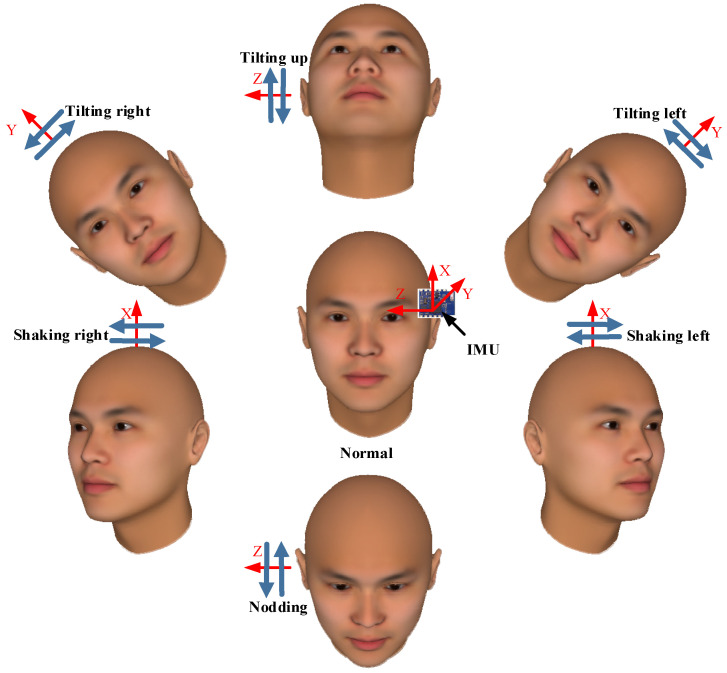
A schematic diagram of the head movement types.

The present study utilized inertial sensors to perceive changes in head posture and collected six types of feature data, including acceleration and angular velocity in the X, Y, and Z directions. The inertial sensor used was the MPU9250, with a sampling frequency of fs = 100 Hz, and was installed on the eyeglass temple close to the front, as shown in the middle view of Figure 1. During data collection, the participants wore glasses equipped with IMU sensors and sat quietly on a stool while naturally performing six different head movements: nodding (Rotation down around the z-axis and recovery to the normal position along the previous motion trajectory, as shown in the bottom view of Figure 1); tilting up (Rotation up around the z-axis and recovery to the normal position along the previous motion trajectory, as shown in the top view of Figure 1); shaking left (Rotation to the left around the x-axis and recovery to the normal position along the previous motion trajectory, as shown in the bottom left view of Figure 1); shaking right (Rotation to the right around the x-axis and recovery to the normal position along the previous motion trajectory, as shown in the bottom right view of Figure 1); tilting left (Rotation to the left around the y-axis and recovery to the normal position along the previous motion trajectory, as shown in the top left view of Figure 1); and tilting right (Rotation to the right around the y-axis and recovery to the normal position along the previous motion trajectory, as shown in the top right view of Figure 1), as shown in the outer view of Figure 1.

The data in this experiment was self-collected using an IMU sensor module with Bluetooth communication. All 25 participants were students from the Zhengzhou University of Light Industry. A total of 25 × 6 datasets were collected and automatically labeled using the head movement activity detection algorithm (detailed in Section 2.2). The accuracy of these labels was verified manually. A total of 2333 data points were labeled, and they were randomly divided into a training set of 1914 and a testing set of 419. The distribution of each type of head gesture is shown in Table 1.

The format of the dataset is *[data, label]*, where *data* is a 6-dimensional matrix consisting of the acceleration and angular velocity data of the x, y, and z-axis collected from the IMU sensor, and the length of the data is variable under the different labels. *Label* is a categorical variable that corresponds to the 6 types of head movements.

### 3.2. Activity Detection for Head Gestures

**Figure 2:**
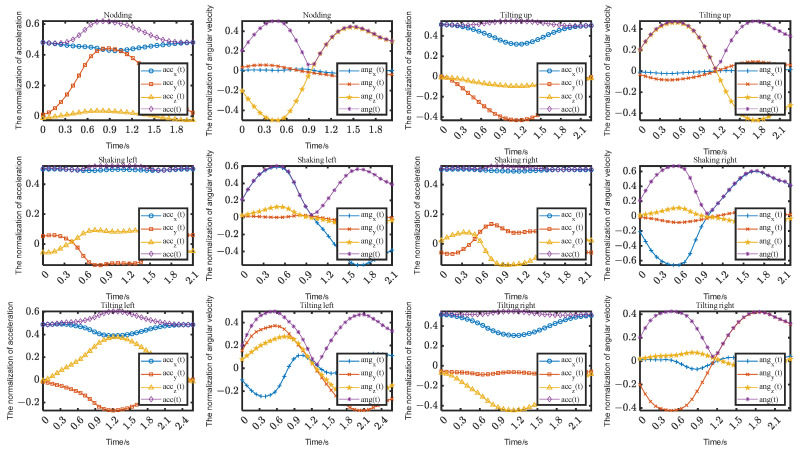
The time series templates for the head movements.

Head pose endpoint detection is essentially distinguished by the different features exhibited during head movements. Based on the acquired data, as shown in Figure 2, it can be observed that the head exhibits similar total angular velocity waveforms regardless of the performed action. Therefore, this paper utilizes this characteristic for the endpoint detection of head actions. The specific detection method is as follows:

(1) Data normalization
  (1) y(t)=arctan(x(t))×2/π
  where y(t) is the normalized data, and x(t) is the collected acceleration and angular velocity data from the sensor.
(2) Sliding median filtering
  (2) $$y(t)=\{\begin{array}{l} \mathrm{median}(x(t-(l-1)/2:t+(l-1)/2)),l=2n-1,n\in N\\ \mathrm{median}(x(t-l/2:t+l/2-1)),l=2n,n\in N \end{array}$$
  where *l* is the length of the filter; l=2n−1,n∈N represents an odd number, and y(t) is the median; l=2n,n∈N represents an even number, and y(t) is the average of the two middle values. The purpose of median filtering is to reduce the salt-and-pepper noise of the sensor and to reduce the possibility of misjudgment in subsequent action recognitions and endpoint detections.
(3) Determining the start time of head movements
  (3) $$\mathrm{if}ang(t)>ang_{min},\exists t_{start}=t$$
  where $ang(t)=\sqrt{ang_{x}^{2}(t)+ang_{y}^{2}(t)+ang_{z}^{2}(t)}$ is the overall description of the angular velocity variation, reflecting the overall degree of change in the angle of head movements. $ang_{x}^{\text{​}}(t)$ $ang_{y}^{\text{​}}(t)$ and $ang_{z}^{\text{​}}(t)$ represent the angular velocity components on the three-dimensional coordinate axes. $ang_{min}$ is the threshold for detecting the start of head movements. $t_{start}$ is the moment when a head movement starts.
(4) Determining the end time of head movements
  (4) $$\mathrm{if}\;(ang(t)<ang_{min})AND(ang(t-t_{interval})<ang_{min}),\exists t_{end}=t$$
  where $t_{interval}$ is a threshold used to determine if a movement has truly ended. Please refer to Figure 2 for details. When the first peak of the head movement ends, the movement is halfway completed. Therefore, it is necessary for the threshold to cross this trough in order to capture the entire waveform of the head movement. Additionally, this threshold also serves as the minimum interval between two consecutive head movements, and $t_{end}$ represents the moment when the head movement ends.
(5) Determining the validity of head movements
  (5) $$\mathrm{if}\;(t_{end}-t_{start}>t_{min})AND(t_{end}-t_{start}>t_{max}),\;\mathrm{There}\;\mathrm{is}\;a\;\mathrm{head}\;\mathrm{movement}\;\mathrm{present}.$$
  where $t_{min}$ is the small duration of the movement used to filter out sharp peak noise in the waveform, and $t_{max}$ is the maximum duration of the movement used to exclude movements with abnormal or incomplete durations.

### 3.3. Head Gesture Recognition Using the DTW Method

The DTW method is a dynamic programming-based algorithm widely used in the fields of speech and posture recognition. This algorithm can warp data on the time axis, stretching or shortening time series to achieve better alignment and improve the accuracy and robustness of the algorithm. Head movements, due to personal habits and different current states, may result in changes in the length of the movement, making it a typical recognition problem of unequal-length time series. Therefore, this paper uses the DTW method to evaluate the warping path distance between different movements and templates to recognize different head movements. The recognition algorithm is as follows:

(1) Calculating time series templates for head movements

To perform action recognition based on the DTW method, a reference template is needed. In order to make this recognition method real-time, an endpoint detection algorithm proposed in this paper is used to extract the time series of the movement. The threshold values used in the algorithm are as follows: *t_win_ =* 0.2 s, *ang_min_* = 0.2 rad/s, *t_min_* = 0.5 s, and *t_max_* = 2.5 s. The effect of other threshold values will be discussed in the experimental analysis. After obtaining the time series for each movement, to effectively evaluate the performance of the algorithm, only the training set is used to create the templates. Taking one movement as an example, suppose a set of data in its time series is $S_{a}=\{s_{1},s_{2},\cdots ,s_{a}\}$, where *a* is the length of this set of data. Then, the total time series set of the training set is $S=\{\overset{n}{\overset{︷}{S_{a},S_{b},\cdots ,S_{k}}}{\}}^{T}$, where *n* is the number of this movement in the training set; *a*, *b*,..., *k* represent the lengths of each sequence; and let the sequence length matrix be $S_{len}=\{a,\;b,\cdots ,k\}$. Therefore, the standard template for this movement is $T=\{t_{1},t_{2},t_{3},\cdots ,t_{m}\}$, where, $t_{i}=\frac{1}{l}\sum_{j=1}^{l}S_{ji}$, l represents the number of occurrences of $S_{ji}$ when *i* is fixed. If $S_{ji}$ does not exist, it is not counted. *m* represents the median of the $S_{len}$ set. By following this process, the standard time series $T_{ij}$ for other head movements can be obtained, where *i* = 1, 2,..., 6 represent six head movements; and *j* = 1, 2,..., 6 represent six types of time series, including acceleration and angular velocity.

(2) Calculating Euclidean distance matrix

Due to the general inability to align standard templates with the time series of the head movements to be recognized, it is clearly unreliable to calculate similarity using linear alignment. In this case, it is necessary to warp a set of time series to align feature points, so that specific feature points are aligned with each other. The specific method is as follows: Let a time series of head movements in the test set be $S=\{s_{1},s_{2},\cdots ,s_{n}\}$ and the time series of the template to be matched be $T=\{t_{1},t_{2},t_{3},\cdots ,t_{m}\}$. Then, the Euclidean distance between any two points is calculated as $d(s_{i},t_{j})=\sqrt{\sum_{k=0}^{5}{\left(s_{ik}-t_{jk}\right)}^{2}}$, forming a n×m distance matrix ***D***. Therefore, the similarity problem between these two time series is transformed into applying dynamic programming to solve the shortest path problem from the starting point ***D***(1,1) to the ending point ***D***(n,m), which is the warping path, denoted by ***W***.

(3) Finding the warping path

The form of the warping path can be represented as follows: $W=\{w_{1},w_{2},w_{3},\cdots ,w_{k}\}$, where $w_{i}$ represents the distance between points ***S*** and ***T***, *k* is the length of the warping path, with a of range max(m,n)≤k≤m+n, and it satisfies the following constraints:

(1) Boundary condition: the path goes from the starting point $w_{1}=D(1,1)$ to the ending point $w_{k}=D(n,m)$.

(2) Continuity: if $w_{i-1}=D(a,b)$, then the next point $w_{i}=D(a^{\prime },b^{\prime })$ on the path needs to satisfy $\left|a-a^{\prime }\right|\leq 1$,$\left|b-b^{\prime }\right|\leq 1$, meaning it cannot skip over a certain point for matching.

(3) Monotonicity: if $w_{i-1}=D(a,b)$, then the next point $w_{i}=D(a^{\prime },b^{\prime })$ on the path needs to satisfy $a^{\prime }-a\geq 0$, $b^{\prime }-b\geq 0$, meaning that for the points on W, the path must monotonically progress over time.

Based on this, we know that there are only three possible paths from $w_{i-1}=D(a,b)$ to the next point: D(a+1,b), D(a+1,b+1), and D(a,b+1). Therefore, the optimal warping path is as follows:

(6) $$\mathrm{DTW}(S,T)=\min\{\frac{1}{k}\sum_{i=1}^{k}w_{i}\}$$

(4) Solving the optima warping path

The dynamic programming idea of the cumulative distance is used to calculate Equation (6). The formula for the cumulative distance is defined as follows:

(7) $$r(i,j)=d(s_{i},t_{i})+\min\{r(i-1,j),r(i-1,j-1),r(i,j-1)\}$$

where i=1,2,3,⋯,n and j=1,2,3,⋯,m. The cumulative distance is actually a kind of recursive relationship, so the best warping path distance between the two time series S,T is DTW(S,T)=r(n,m). This solves the problem of measuring the similarity of time series with different lengths and unaligned feature positions.

(5) Determining the type of head movement

To separately calculate the DTW values between the head movement time series and the time series of the 6 template movements, repeat the above steps, and the minimum value will then represent the type of this movement.

## 4. Experimental Results and Discussion

### 4.1. Experimental Platform

This experiment’s hardware environment uses an Intel i7-8700 processor with 16 GB of memory, a P2000 graphics card with 5 GB of memory, Bluetooth 4.0 communication, and a sensor chip MPU9250 with a sampling frequency of 100 Hz.

### 4.2. Selection of Endpoint Detection Parameters

**Figure 3:**
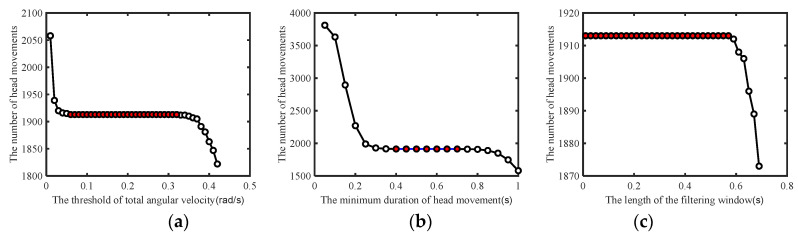
The impact of parameters on endpoint detection. (**a**) The impact of angular velocity threshold on the number of head motion recognition. (**b**) The impact of minimum head motion duration on the number of head motion recognition. (**c**) The impact of filtering time window length on the number of head motion recognition. In these figures, the areas filled with red inside the circles represent the thresholds that can correctly recognize the number of head movements, otherwise it is inaccurate.

Based on the head movement template in Figure 2, it can be observed that head movements generally exhibit a back-and-forth motion, which is manifested as two peaks in the angular velocity *ang_min_*. In order to correctly identify these two peaks, it is necessary to determine the threshold of the angular velocity. At a minimum duration of head movement *t_min_* = 0.5 s, the threshold of the angular velocity was tested in the range of [0.01, 0.42] with an increment of 0.01, and it was found that correct endpoint detection can be achieved within the range of [0.06, 0.32] of the angular velocity; hence, *ang_min_* = 0.2 was selected, as shown in Figure 3a. Subsequently, at *ang_min_* = 0.2 rad/s, the minimum duration of head movement was tested in the range of [0.05, 1] with an increment of 0.05, and it was found that correct endpoint detection can be achieved within the range of [0.4, 0.7]; hence, *t_min_* = 0.6 s was selected, as shown in Figure 3b. Finally, the influence of the median filtering length on the results was examined, taking *ang_min_* = 0.2 rad/s and *t_min_* = 0.6 s, resulting in *t_win_* = 0.3 s, as shown in Figure 3c.

### 4.3. An Analysis of the Recognition Effectiveness of the DTW Algorithm

To verify the accuracy of the DTW algorithm for head movement recognition, head movement templates were created using a training set, and the effectiveness of the algorithm was tested using a test set. The DTW algorithm was used to calculate the DTW values corresponding to the shortest path, reflecting the similarity of the two curves, i.e., the quality of the match. The better the match between the two curves, the higher the similarity and the shorter the shortest warping path, resulting in a smaller DTW value. Conversely, the worse the match between the two curves, the less similar the matching curves and the longer the shortest warping path, resulting in a larger DTW value. Ideally, when two matching curves are completely aligned, the shortest warping path is 0, meaning the DTW value is 0. In order to distinguish between these six types of head movement characteristics, this study, respectively, weighed the DTW values of the six curves, with the smallest DTW value corresponding to the specific head movement. The results show a recognition accuracy of 100%.

To demonstrate the effectiveness of this method, this paper uses an LSTM network as a control algorithm. Convolutional neural networks, as representatives of feedforward neural networks, have shown significant performance in classifying fixed-length spatial data, such as images and videos. However, for the processing of sequential data related to human pose detections, the effect is not too ideal due to the constraints of its own network structure. The LSTM network belongs to the recurrent neural network and has memory, which can model the temporal sequence of data and has a good fitting effect on sequential data. In the field of deep learning, traditional feedforward neural networks perform well in many aspects. Therefore, this paper adopts an LSTM neural network, with detailed parameters, as shown in Table 2. Specifically, the initialization method of batch size weights is very important for training neural network models. In this paper, the orthogonal initialization is chosen to ensure that the initial weights are orthogonal, in order to solve the problems of vanishing gradients and exploding gradients that can occur during the training process of an LSTM network. The training and test data are consistent with the DTW method, and after verification, the accuracy is only 86.31%.

A further analysis revealed that although the LSTM method has a significantly lower recognition rate compared to the DTW method, its recognition speed is higher than the DTW method. This is because the DTW method requires calculating the similarity of six sequences and comparing their magnitudes. However, the training time for an LSTM is much higher than that of the DTW method.

## 5. Conclusions

This paper presents a head movement detection method based on DTW. It collects feature data of head movement postures, including the acceleration and angular velocity in the X, Y, and Z directions, through an IMU fixed on the glasses. The method extracts the motion interval of head movements using endpoint detection and constructs a head movement template. Subsequently, it calculates the optimal path between the head movement data and the head movement template data, where the minimum value corresponds to the standard template of the head movement type, thus determining the head movement type of the test data. Compared to previous studies, the method proposed in this paper uses endpoint detection to automatically segment the head motion time series and accurately recognize the head motion types of the subjects using six features based on DTW. This provides a new option for human–computer interface technology in the field of head gesture recognition. Additionally, it is cost-effective, requires minimal data processing, provides quick responses, and achieves high recognition accuracy.

## Figures and Tables

**Table 1:** Head gesture classification dataset.

Head Movement Types	Number of Training Samples	Number of Testing Samples
Nodding	293	67
Tilting up	317	69
Shaking left	326	64
Shaking right	302	71
Tilting left	337	80
Tilting right	339	68
Total	1914	419

**Table 2:** The parameters of the LSTM neural network.

Parameters	Value	Parameters	Value
Hidden layer	2	Loss function	Cross entropy
Number of hidden layers, 1 unit	200	Learning rate	0.0001
Number of hidden layers, 2 units	100	Batch size	128
Maximum number of iterations	1000	The initialization method of batch size weights	Orthogonal initialization

## Data Availability

The data that support the findings of this study are available from the corresponding author upon reasonable request.
